# Prospective, Randomized, Double‐Blind, Placebo‐Controlled Study of an Oral Antioxidant‐Rich Synbiotic Supplement on Skin Health and Photoaging

**DOI:** 10.1111/jocd.70836

**Published:** 2026-04-07

**Authors:** Laila Afzal, Nhi Nguyen, Nasima Afzal, Sarah Adnan, Zillehuma Khan, Ajay S. Dulai, Subhendu Nayak, Rishi R. Trivedi, Asher Min, Raja K. Sivamani

**Affiliations:** ^1^ Integrative Skin Science and Research Sacramento California USA; ^2^ Integraive Research Institute Sacramento California USA; ^3^ Vidya Herbs Florida USA; ^4^ Pacific Skin Institute Sacramento California USA; ^5^ Department of Dermatology University of California‐Davis Sacramento California USA; ^6^ College of Medicine California Northstate University Elk Grove California USA

**Keywords:** antioxidant, elasticity, herbs, hydration, photoaging, skin, synbiotic

## Abstract

**Background:**

Photoaged skin is primarily induced by prolonged exposure to ultraviolet radiation, leading to increased pigmentation, reduced elasticity, and pronounced wrinkles. There has been an increasing recognition of the influence of nutrition on skin health, with dietary elements emerging as a viable alternative approach to preventing photoaging. This study explores how oral supplementation impacts photoaged skin, hydration, and elasticity.

**Aims:**

This study evaluates the effects of an antioxidant‐rich synbiotic supplement on facial elasticity, hydration, and photoaging markers, such as erythema and pigmentation.

**Patients/Methods:**

In this 8‐week study, 36 participants from the Sacramento region were randomized to receive either the antioxidant‐rich synbiotic supplement or a placebo. Participants had 3 visits: baseline, week 4, and week 8. Noninvasive facial measurements, including hydration, elasticity, erythema, and pigmentation, were obtained. Facial photography and analysis were performed. Tolerability was assessed.

**Results:**

At week 8, the treatment group showed a significant reduction in wrinkle severity (−5.28%, *p* < 0.01), whereas the placebo group did not. Cheek elasticity increased significantly at week 4 (22.0%, *p* < 0.01) and week 8 (13.8%, *p* < 0.05), along with firmness at week 8 (12.2%, *p* < 0.01). The placebo group showed no significant changes in these parameters. Additionally, the viscoelasticity of the cheek increased at week 4 (30.2%, *p* < 0.01) in the treatment group, with no change in the placebo group.

**Conclusions:**

The antioxidant‐rich synbiotic supplement improved wrinkle severity, skin facial elasticity, and skin firmness, enhancing several parameters of photoaging.

## 1 Introduction

Human skin is a complex organ that covers the entire human body. It undergoes both chronological aging and photoaging [1]. Photoaging refers to the environmental impact on skin over time [1] and is primarily impacted by the amount of skin pigment and sun exposure [2]. Aging skin can exhibit uneven skin tone, wrinkles, thinning, and a loss of elasticity [3]. Accumulation of damage leads to increased reactive oxygen species and changes the properties and quantity of matrix proteins [4]. Collagen, elastin, and keratin are proteins found in the skin. Collagen prevents skin aging, is anti‐inflammatory, and promotes wound healing [4]. It is crucial to skin structural support and quality and is compromised by environmental factors [3]. Reduced collagen has been shown to contribute to skin aging and is what contributes to wrinkle formation [4].

Defense against photoaging historically relied upon topical therapy, but there has been a greater understanding that the “inside‐out” approach with the use of supplements is important for photoprotection. For example, studies with *Polypodium leucotomas*, oral carotenoids, vitamin E, and vitamin D supplementation have shown photoprotective abilities [5]. Studies with probiotic supplementation have shown promising results in animal studies [6], although probiotic supplementation studies in humans for photoaging are needed.

Synbiotics are supplements composed of probiotics (microorganisms) and prebiotics (food for microorganisms) that can work synergistically to benefit the host [7]. This study evaluates an antioxidant and synbiotic formulation on photoaging with a mix of *Bacillus‐based* probiotics, Konjac glucomannan (KGM), and astaxanthin, among other components. Astaxanthin (ASX) is an α‐hydroxy‐ketocarotenoid found in numerous bacterial species, microalgae, and marine animals, including shrimp, crab, and salmon [8, 9]. Because of its pigment‐producing properties, it is standardly used in feed for various fish and animal feed [10]. It is a valuable nutraceutical with potent antioxidant effects [11]. Additionally, it has been shown to influence human health, including the aging process [10]. It has demonstrated anti‐inflammatory, potent antioxidant, and skin‐protective effects [12]. In the skin ASX can reduce wrinkle severity, reduce hyperpigmentation, and combat photoaging by protection from ultraviolet (UV) damage by supporting DNA repair, and accelerating wound healing [13, 14].

KGM is a water‐soluble polysaccharide derived from the konjac plant ( *Amorphophallus konjac* ) [15]. The molecular structure of KGM consists of β–glucose and β–mannose, with a minor amount of acetyl groups, and it has a chain–like structure with a state of random curl [16]. Its unique structure gives it the ability to increase water absorption, and it is typically used as a food additive [17]. Additionally, it is widely used as a wound healing dressing. It has antibacterial properties and can provide a moist environment that is optimal for wound healing [16]. As a nutritional supplement, it has been effective in treating obesity, constipation, and diabetes. In the skin, it combats photoaging through skin cell regeneration and has been used to treat acne [18]. In one study, it prevented skin inflammation and hyper‐ IgE production in mice with atopic dermatitis through downregulation of IFN‐γ [19].

*B. coagulans, B. subtilis, and B. clausii* are all spore‐forming *Bacillus* bacteria, *with B. coagulans* noted to be a lactic‐acid‐*producing* Bacillus that has been shown to modulate the gut‐skin axis and improve the appearance of skin [20]. Probiotics decrease serum excretion rate in both patients with and without acne, have been shown to increase transepidermal water loss, and decrease lesion count in acne patients [20].

In this study, we assess the efficacy of an ASX‐rich synbiotic supplement on skin health and markers of photoaging, including elasticity, hydration, erythema, and pigmentation.

## 2 Materials and Methods

### 2.1 Investigational Products

The active study product consisted of the following active ingredients: 5 mg Astaxanthin, 100 mg Skin Cera, 25 mg *B. coagulans* , 25 mg *B. subtilis* , and 15 mg *B. clausii*. These ingredients were diluted in 112 mg fructooligosaccharides (FOS), 6 mg Medium‐Chain Triglycerides (MCT) powder, 6 mg Nuflow, and 6 mg Numag. The placebo product consisted of 288 mg microcrystalline cellulose (MCC), 6 mg Nu‐FLOW, and 6 mg Nu‐MAG. Nu‐FLOW is an anti‐caking agent that prevents the powder from clumping in the capsule [21]. Nu‐MAG is an organic lubricant that improves powder flow and reduces friction when tableting [22]. Because of the photosensitive nature of the astaxanthin, both the synbiotic and the placebo capsules were placed in opaque bottles that were protected from light exposure. Both the synbiotic and placebo were supplied by Vidya Herbs (Bangalore, Karnataka, India). Subjects were instructed to take one capsule of their assigned supplement once daily.

### 2.2 Subjects

This 8‐week double‐blinded randomized clinical trial was conducted from January 2024 to June 2024 at Integrative Skin Science and Research in Sacramento, California. The study was approved by the Allendale Institutional Review Board on November 9, 2023, and was registered on clinicaltrials.gov (NCT06146140). Participants for this study were recruited from the greater Sacramento area. All participants provided written informed consent prior to enrollment in the study. Subjects were randomized into two groups: the active product or the placebo, utilizing a computer‐based randomization generator. The randomization was allocated a priori using sealed, blinded envelopes. Investigators, clinical research coordinators, and participants were all blinded to the study intervention.

### 2.3 Inclusion and Exclusion Criteria

The inclusion criteria for this study were women aged 35–55 years old presenting with facial fine lines and wrinkles. Exclusion criteria included: individuals who are pregnant or breastfeeding, prisoners, individuals unable to consent, individuals who have used topical probiotics, topical hydroquinone, retinoids, bakuchiol, vitamin C, or acetyl zingerone containing products in the past 2 weeks, individuals who have undergone any medical treatment designed to improve the appearance of facial skin in the past 3 months or who plan to undergo such treatment, individuals who have changed any hormonal based contraception within the past 3 months, individuals that currently smoke tobacco or have a tobacco smoking history of greater than 10 pack years, individuals with allergies to any study product ingredients, individuals who have utilized an oral antibiotic within the past 1 month, and individuals unwilling to discontinue oral probiotics‐based supplementation or supplement ingredients located in the study product for 1 month prior to enrollment.

### 2.4 Study Visits and Procedures

This study consisted of 4 visits: a screening visit to determine subject eligibility, a baseline visit (week 0), a follow‐up visit (week 4), and a final visit (week 8). At week 0, subjects were randomized and instructed to start using the study product. High‐resolution photographs of the face were captured using the BTBP 3D Clarity Pro Facial Modeling and Analysis System [23] (Brigh‐Tex BioPhotonics, San Jose, CA, USA) to analyze facial wrinkles, fine lines, skin texture, and erythema at weeks 0, 4, and 8. Skin biophysical measurements were performed on the cheeks at weeks 0, 4, and 8, using the Cutometer (Courage and Khazaka, Köln, Germany) to measure skin firmness and elasticity, the MoistureMeterSC (Delfin Technologies, Stamford, CT, USA) to measure skin hydration, and the VapoMeter (Delfin Technologies, Stamford, CT, USA) to measure transepidermal water loss. A digestive questionnaire was given out at weeks 0, 4, and 8. A subjective questionnaire to measure skin appearance including skin texture, pigment, elasticity, feel, and erythema was given at weeks 4 and 8. The subjective questionnaire was ranked on a Likert scale from 0–5 with 0 meaning strongly agree and 5 meaning strongly disagree.

### 2.5 Statistical Analysis

Two‐tailed Student's *t*‐tests were used to compare week 0 (baseline) measurements to those at week 4 and week 8, with statistical significance set at *p* ≤ 0.05. Results were presented as mean and standard error of the mean. Data collected at week 0 served as the control for comparisons with week 6 data. Data visualization was performed using Prism v. 10 (GraphPad Software LLC, San Diego, CA, USA).

## 3 Results

**FIGURE 1:**
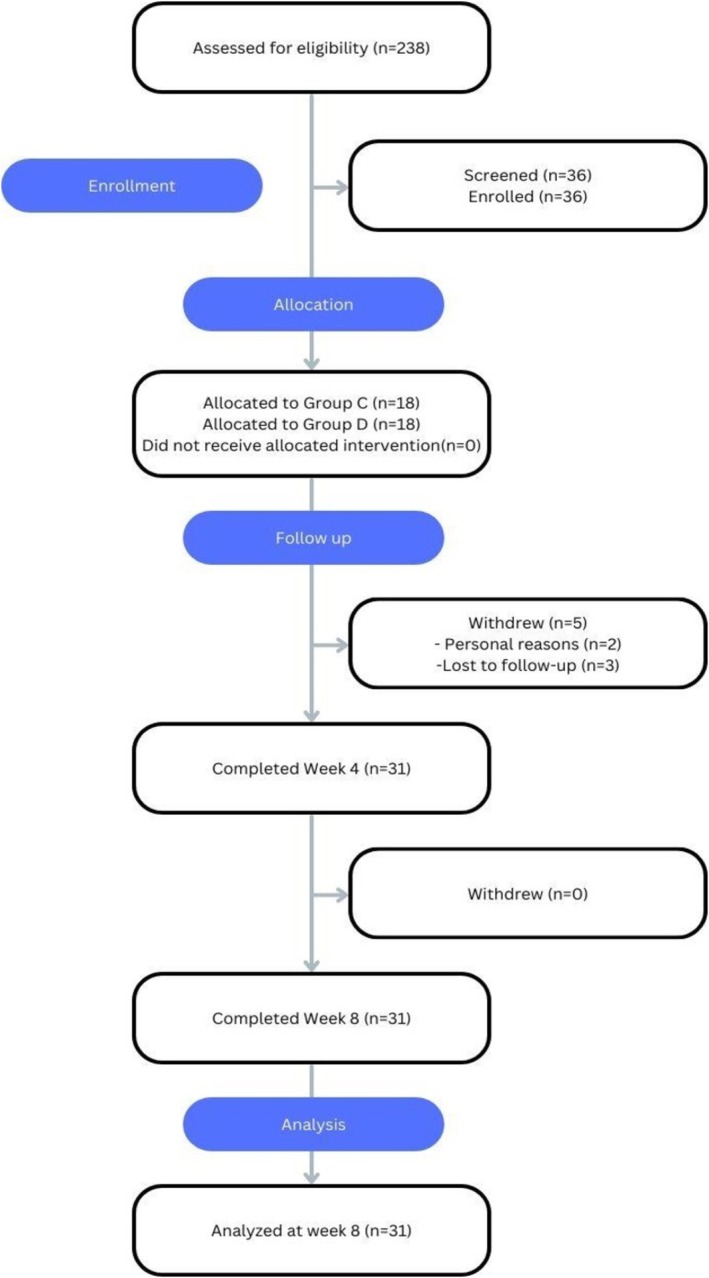
CONSORT (Consolidated Standards of Reporting Trials) flow diagram.

36 subjects met all inclusion/exclusion criteria and were enrolled to begin the 8‐week treatment phase, and 31 subjects completed the study (Figure 1). The average age of enrolled subjects was 48 years old (± 7), and the cohort consisted of 36 females (100%). Fitzpatrick skin types (FST) of the cohort included FST 1 [3], FST 2 [7], FST 3 [16], FST 4 [8], FST 5 [1].

### 3.1 Wrinkle Severity

**FIGURE 2:**
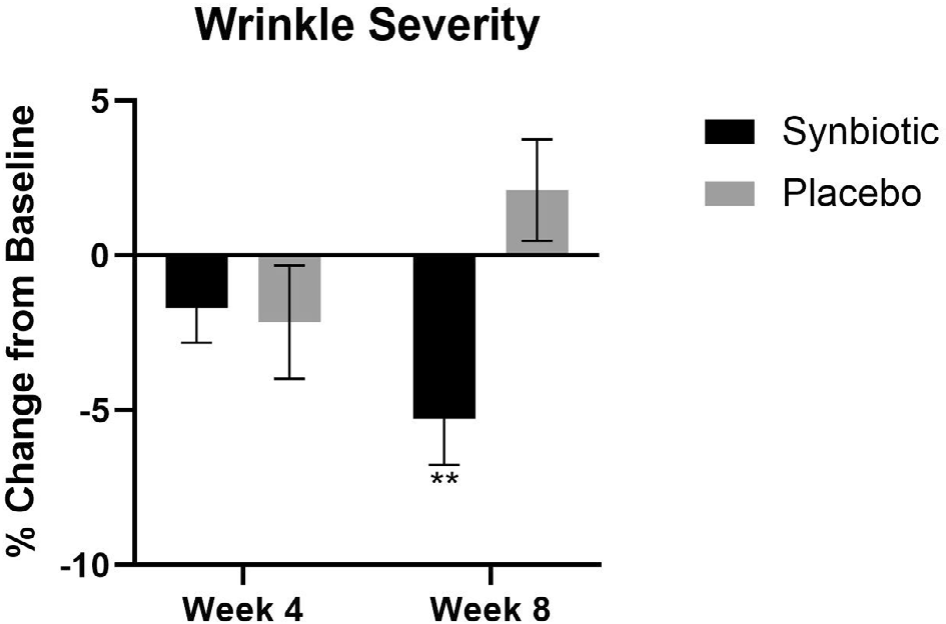
Wrinkle severity percent change in the active group and the placebo group at week 4 and week 8 compared to their baseline. **p < 0.01.

The synbiotic group had a decrease in the wrinkle severity at week 8 (−5.3%, *p* = 0.003) and there was no significant change in wrinkles in the placebo group (Figure 2).

### 3.2 Facial Elasticity Measures

#### 3.2.1 Firmness (R0)

**FIGURE 3:**
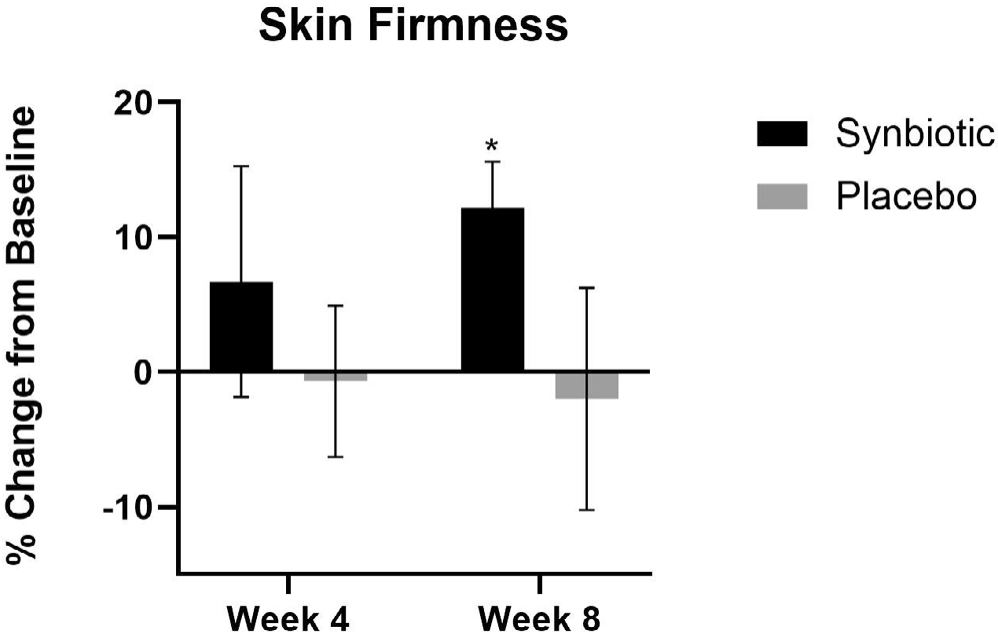
Wrinkle severity percent change in the active group and the placebo group at week 4 and week 8 compared to their baseline. *p < 0.01.

At week 4, there were no statistically significant changes in skin firmness, but at week 8 there was a 12.2% increase in the firmness (*p* < 0.01). There were no significant changes in firmness in the placebo group (Figure 3).

#### 3.2.2 Net Elasticity (R5)

**FIGURE 4:**
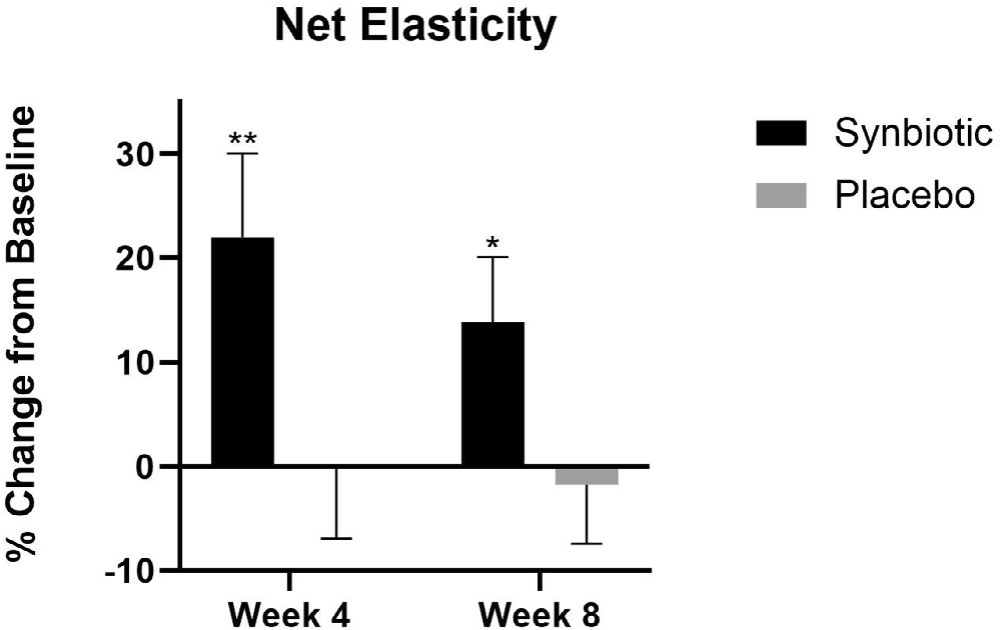
Net elasticity percent change at week 4 and week 8 compared to their baseline. **p < 0.01, *p < 0.05.

There was a significant increase in facial skin net elasticity at week 4 (22.0%, *p* < 0.01) and an increase at week 8 (13.8%, *p* < 0.05) compared to baseline in the synbiotic group. There were no significant differences in the placebo group (Figure 4).

#### 3.2.3 Viscoelasticity (R2)

**FIGURE 5:**
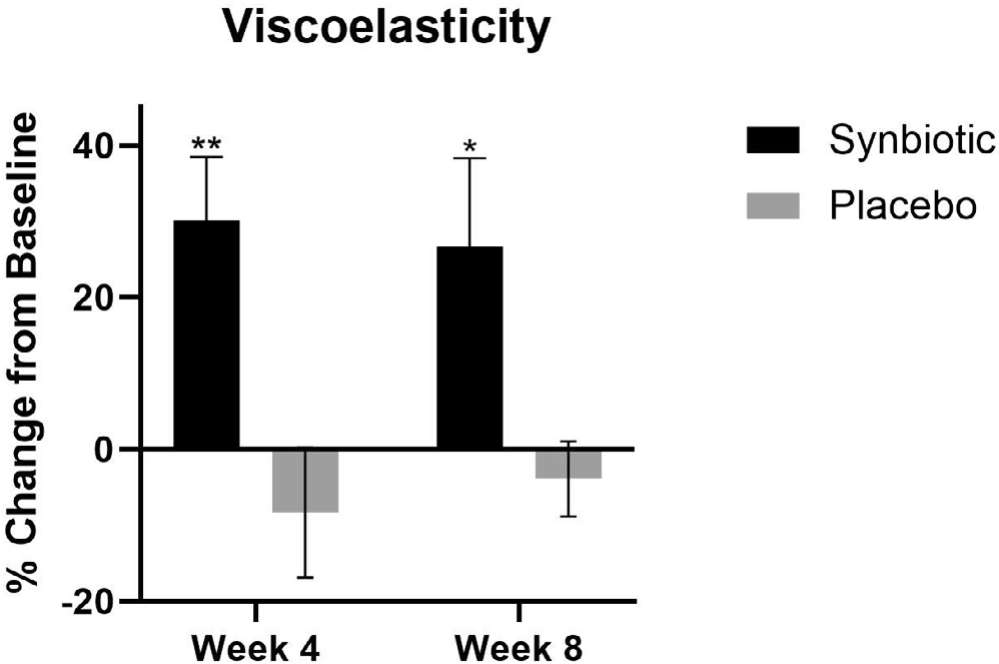
Viscoelasticity percent change in the synbiotic group (active) and the placebo group at week 4 and week 8 compared to their baseline. *p < 0.05, **p < 0.01.

The viscoelasticity was increased at both week 4 (30.2%, *p* < 0.01) and at week 8 (26.7%, *p* < 0.05) compared to baseline. There were no significant changes in the viscoelasticity in the placebo group (Figure 5).

### 3.3 Erythema Intensity

**FIGURE 6:**
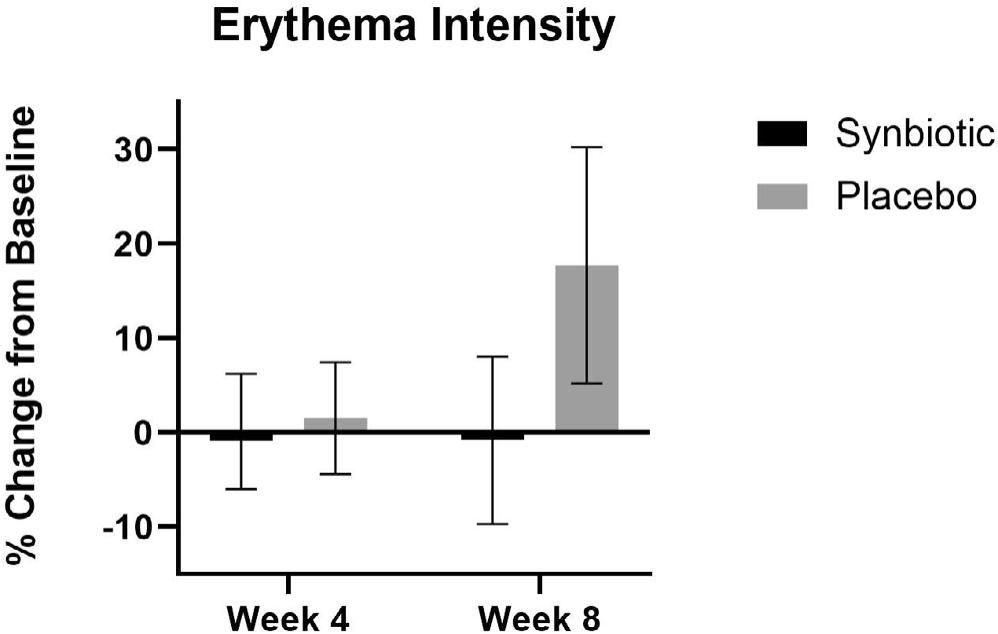
Erythema intensity percent change in the synbiotic (active) group and the placebo groups at week 4 and week 8 compared to their baseline.

The synbiotic and the placebo groups did not have any statistically significant differences from baseline (Figure 6).

### 3.4 Skin Hydration

**FIGURE 7:**
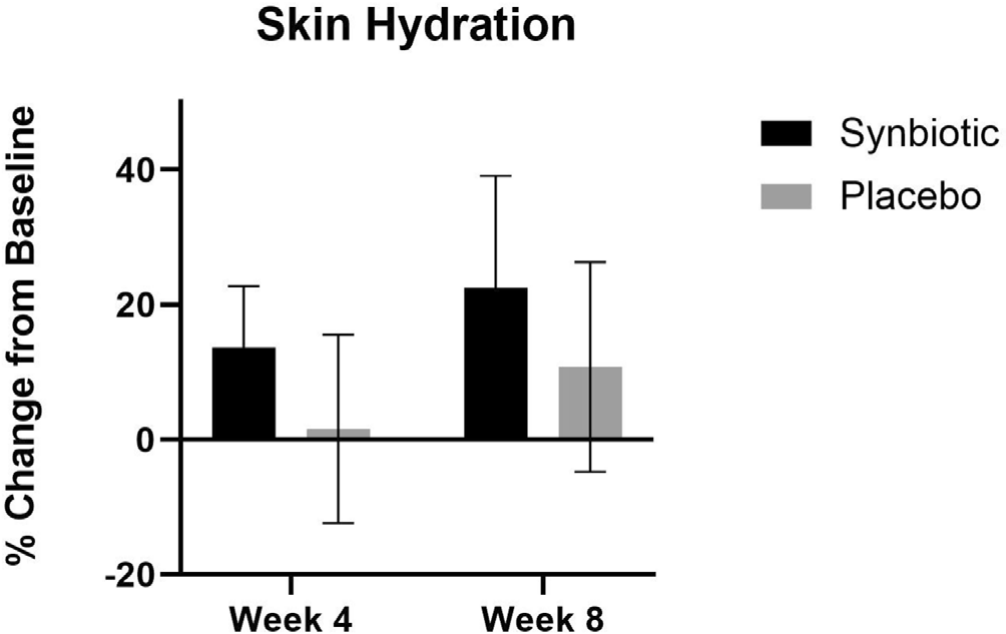
Skin hydration percent change in the active group and the placebo group at week 4 and week 8 compared to their baseline. None of the changes were statistically significant.

At week 4 and week 8, there was no significant increase in facial hydration in either treatment group (Figure 7).

### 3.5 Transepidermal Water Loss

**FIGURE 8:**
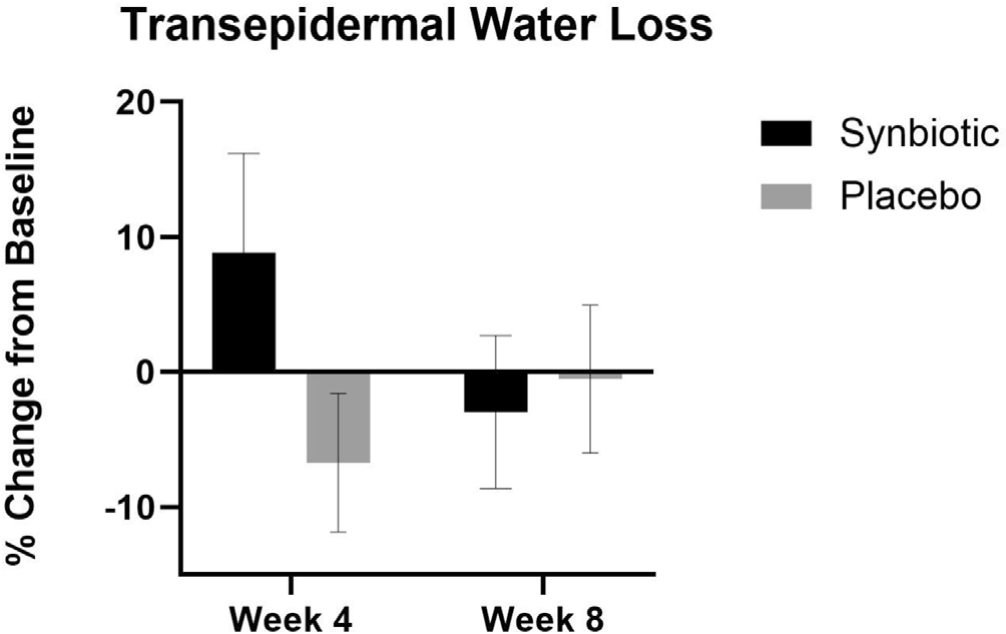
TEWL percent change in the active group and the placebo group at week 4 and week 8 compared to their baseline. None of the changes were statistically significant.

At week 4 and week 8, there was no significant increase in facial transepidermal water loss in either treatment group (Figure 8).

### 3.6 Facial Photography

**FIGURE 9:**
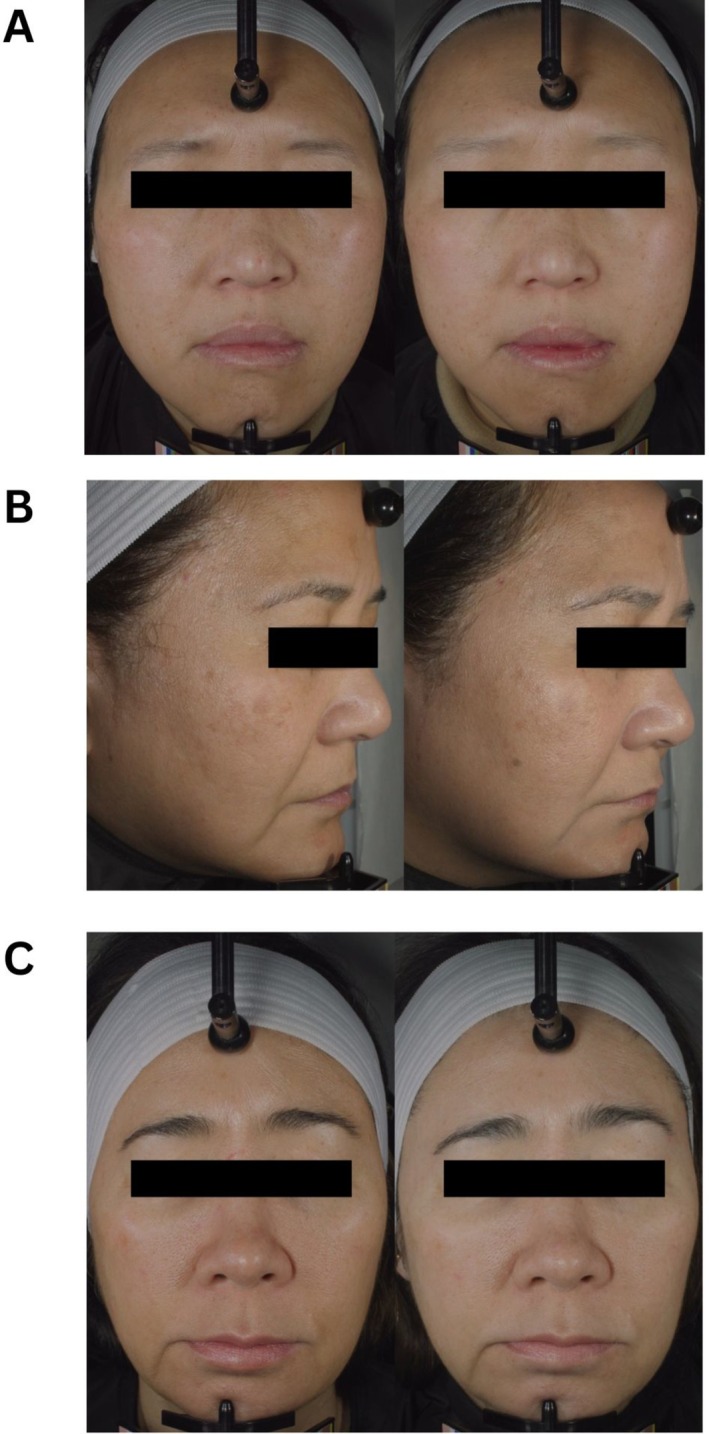
(A) Synbiotic group subject at week 0 (left) and week 8 (right). (B) Synbiotic group subject at week 0 (left) and week 8 (right). (C) Synbiotic group subject at week 0 (left) and week 8 (right).

High resolution facial photography was taken for both intervention groups at week 0 (baseline), week 4, and week 8 (Figure 9).

### 3.7 Adverse Events

There were no adverse events throughout the study in either group.

## 4 Discussion

This 8‐week study showed that supplementation with an astaxanthin‐rich synbiotic improved markers of photoaging, including the appearance of wrinkles as well as skin biomechanical factors such as skin pliability/firmness, elasticity, and viscoelasticity. The use of probiotics has grown over the past decade, but there are few reports of the use of probiotics for photoaging. This study serves as one of the few studies that have evaluated the use of spore‐based probiotics as part of a synbiotic formulation.

Overall, the findings reported here are supported by previous work that has evaluated the individual ingredients. For example, ASX may aid in improving markers of photoaging because of its potent antioxidant and anti‐inflammatory activity [24]. Matrix metalloproteinase‐1 (MMP‐1) is a collagenase that breaks down type I collagen, type III collagen, and elastin in the skin's dermis. UV irradiation stimulates inflammatory and oxidative pathways, which lead to increased secretion of MMP‐1 by dermal fibroblasts [25]. One study demonstrated that oral supplementation of ASX dose‐dependently suppressed inflammatory cytokines and MMP‐1 in UVB‐irradiated keratinocytes [26]. Another study demonstrated that oral ASX supplementation protected against UV‐induced decrease of moisture and increased minimal erythema dose (MED) in healthy adults [9]. Limitations to ASX use include its high‐cost production process, which will potentially reduce as scientists develop novel methods of bio‐refinery [27, 28].

KGM induces the growth of new cells after UVB‐induced cellular damage [17]. Specific benefits of konjac in the skin include the reduction of redness, dryness, hyperpigmentation, itching, and oiliness [29]. This acts as a protective mechanism against UV‐induced signs of photoaging, which are a result of degradation of collagen, fibronectin, elastin, and proteoglycans, and increased oxidative stress [30].

Three species of *Bacillus* were included in this oral supplement. *Bacillus* is spore‐forming, meaning that it demonstrates excellent stability and the capability to withstand the gastric system [31]. *B. coagulans* oral administration has demonstrated increased levels of hyaluronic acid and IL‐10 [32]. Hyaluronic acid is one of the major components of our skin associated with skin hydration which can be degraded by reactive oxygen species [33]. Current evidence strongly links hyaluronic acid to improved signs of photoaging, including wrinkles [34]. Oral supplementation of *B. subtilis* has been shown to reduce TNF‐α [35, 36]. TNF‐α is an inflammatory cytokine that leads to the production of matrix metalloproteinases that degrade collagen and elastin fibers [37, 38]. This degradation of these fibers has been shown to cause photoaging, including increased wrinkle severity and loss of elasticity within the skin [39, 40].

Notably, the action of prebiotics can synergistically improve the efficacy of probiotics. Prebiotics can stimulate healthy bacteria through the action of fermentation [41]. FOS has been well researched to demonstrate increased abundance of *Bifidobacterium* and *Lactobacillus* [42]. Notably, these two species have demonstrated in vitro stimulation of tight‐junction proteins such as occludin and claudin1, which can be protective against altered tight junction protein expression involved in photoaging [43, 44]. Further, konjac has demonstrated prebiotic properties, which can further potentiate the effects of the included *Bacillus* in this oral supplement [45].

There has been increasing interest in the use of synbiotics for the synergistic potential of combining probiotics with antioxidants and prebiotics [46]; however, there are few studies evaluating the use of synbiotics for skin health and photoaging. Our study shows clinical evidence for using synbiotics for photoaging and also offers some mechanistic insights. The changes in skin elasticity measures, along with wrinkles, suggest that changes in the collagen and elastin content may be contributing to the changes seen with wrinkles. Additionally, the role of matrix metalloproteinases should be further explored to understand how the balance of collagen and elastin synthesis and the breakdown of collagen are modulated by the supplement. Future studies with histological analysis for changes in collagen, elastin, and matrix metalloproteinases are warranted.

This study was a pilot study with a small group of participants. However, each participant was analyzed as their own control, and objective measures with high resolution were utilized, which increases the power of the study. Overall, the results noted here warrant future studies with an expanded population and may incorporate combination therapies with both supplementation and topically based therapies.

## 5 Conclusions

In conclusion, our clinical trial findings collectively suggest that an antioxidant‐rich synbiotic intervention supports skin health by increasing skin facial elasticity and firmness and reducing wrinkle severity. The trial findings are significant for the skincare industry, suggesting that this intervention may provide an effective oral supplementation solution for improving cosmetic appearance and managing signs of aging. The promising results of this study lay the groundwork for future research, which may ultimately enhance our understanding of its utility in the skincare and dermatology field.

## Author Contributions

Conceptualization, Raja K. Sivamani; data curation, Laila Afzal, Nhi Nguyen, Nasima Afzal, Sarah Adnan, Zill‐e‐huma Khan, Ajay S. Dulai, and Asher Min; formal analysis, Laila Afzal, Nhi Nguyen, Nasima Afzal, Zill‐e‐huma Khan, Ajay S. Dulai, Asher Min, and Raja K. Sivamani; funding acquisition, Subhendu Nayak and Rishi R. Trivedi; investigation, Laila Afzal, Nhi Nguyen, Nasima Afzal, Sarah Adnan, and Raja K. Sivamani; methodology, Raja K. Sivamani; project administration, Raja K. Sivamani; resources, Raja K. Sivamani; supervision, Raja K. Sivamani; validation, Raja K. Sivamani; visualization, Raja K. Sivamani; writing – original draft, Laila Afzal, Nasima Afzal, Zill‐e‐huma Khan, Ajay S. Dulai, and Asher Min; writing – review and editing, Laila Afzal, Nasima Afzal, Zill‐e‐huma Khan, Ajay S. Dulai, Asher Min, and Raja K. Sivamani.

## Funding

This work was supported by Vidya Herbs.

## Ethics Statement

The study, # i23_Vidya_Skin_Supp, was conducted according to the guidelines of the Declaration of Helsinki and approved by the Allendale IRB on 11/09/2023, and all participants provided written informed consent and written photo consent prior to participation.

## Conflicts of Interest

Raja K. Sivamani is a consultant for Arbonne, Trace Minerals, Codex Labs, Burt's Bees, Image Skincare, Novartis, Sanofi, Bristol Myers Squibb, Pfizer, Nutrafol, Lilly, Arcutis, Galderma, Incyte, AbbVie, Leo, UCB, Sun, and Regeneron Pharmaceuticals.

## Data Availability

The data are available from the corresponding author on reasonable request.
